# A HIV-1 heterosexual transmission chain in Guangzhou, China: a molecular epidemiological study

**DOI:** 10.1186/1743-422X-6-148

**Published:** 2009-09-25

**Authors:** Zhigang Han, Tommy WC Leung, Jinkou Zhao, Ming Wang, Lirui Fan, Kai Li, Xinli Pang, Zhenbo Liang, Wilina WL Lim, Huifang Xu

**Affiliations:** 1 Guangzhou Center for Disease Control and Prevention, Guangdong, PR China; 2 Virology Division, Public Health Laboratory Services Branch, Center for Health Protection, Hong Kong SAR; 3 Jiangsu Provincial Center for Disease Control and Prevention, Nanjing, PR China; 4 Huadu Center for Disease Control and Prevention, Guangdong, PR China

## Abstract

**Background:**

We conducted molecular analyses to confirm four clustering HIV-1 infections (Patient A, B, C & D) in Guangzhou, China. These cases were identified by epidemiological investigation and suspected to acquire the infection through a common heterosexual transmission chain.

**Methods:**

*Env C2V3V4 *region, *gag p17/p24 *junction and partial *pol *gene of HIV-1 genome from serum specimens of these infected cases were amplified by reverse transcription polymerase chain reaction (RT-PCR) and nucleotide sequenced.

**Results:**

Phylogenetic analyses indicated that their viral nucleotide sequences were significantly clustered together (bootstrap value is 99%, 98% and 100% in *env*, *gag *and *pol *tree respectively). Evolutionary distance analysis indicated that their genetic diversities of *env*, *gag *and *pol *genes were significantly lower than non-clustered controls, as measured by unpaired *t*-test (*env *gene comparison: *p *< 0.005; *gag *gene comparison: *p *< 0.005; *pol *gene comparison: *p *< 0.005).

**Conclusion:**

Epidemiological results and molecular analyses consistently illustrated these four cases represented a transmission chain which dispersed in the locality through heterosexual contact involving commercial sex worker.

## Background

Epidemiology of human immunodeficiency virus type 1 (HIV-1) infection in China is changing from predominantly injecting drug use to increasingly sexual transmission [1], with close to 50% of new infections attributable to sexual transmission in 2005 [2]. The unsafe sexual behavior is one of the key risk factors of HIV transmission in China. A review cited the Newsweek reported that the number of commercial sex workers (CSWs) in mainland China exceed 10 million in 2003 [3]. Data from a nation-wide sentinel surveillance revealed that HIV prevalence among CSWs has risen from 0.02% in 1996 to 0.93% in 2004 and over 1% in some places [4]. Recently, a comprehensive surveillance revealed that 60% of CSWs do not use condoms every time [1]. These results suggested that CSWs serve as a bridge to transmit HIV from core risk groups into general population of the country.

What puzzles epidemiologists is the difficulties in defining the linkage amongst HIV cases using traditional epidemiological approaches based on behavioral information. Nucleotide sequence analysis presents an unique opportunity to identify possible epidemiological linkage between infected cases and to track the viral transmission from person to person through viral genome analysis, as explored in various HIV-1 studies [5,6].

We conducted molecular analyses to confirm four HIV-1 infections from Guangzhou China which were transmitted through a heterosexual transmission chain involving CSW.

## Methods

### Patients, materials and methods

Serum specimens from four patients (Patients A, B, C and D) and five contacts (Contacts 1, 2, 3, 4 and 5) in this surveillance investigation were collected between January 2008 and May 2008 after obtaining their informed consent. These sera were collected prior to any antiretroviral treatment. Sera were screened for HIV-1 antibody by enzyme-linked immunosorbent assays (bioMerieux, France and Peking BGI-GBI, China) and confirmed by Western blotting (MP Biomedicals, Singapore). HIV-1 positive sera were aliquoted to avoid unnecessary freeze-thaw deterioration cycle and were stored at -70°C.

### RNA extraction/PCR/nucleotide sequencing

RNA extraction, reverse transcription polymerase chain reaction (RT-PCR) amplification and nucleotide sequencing were performed in physically separated laboratories. Viral RNA was extracted by QIAamp Viral RNA extraction Mini kit (Qiagen, Hilden, Germany) according to the manufacturer's instructions. Extracted RNA was reverse transcribed by MuLV reverse transcriptase (Applied Biosystems, Inc., Foster City, CA) into cDNA using random hexamer. The cDNA was used as template for PCR amplification of two HIV-1 subgenomic regions (*env C2V3V4 *region and *gag p17/p24 *junction) by nested PCR. In addition, the cDNA from four clustered cases was further amplified at the *pol *gene. The primers and conditions of PCR for *gag *and *env *applied in this study were as previously described [7]. The region of *pol *gene was divided into three segments (*pro*, *rt5' *and *rt3'*). The primers and conditions for *pol *were as follows: *pro *segment: outer sense primer (PolF20): 5'-GAG AGA CAG GCT TAT TTT TT-3', common antisense primer (PR96): 5'-CTT CCC AGA AGT CTT GAG TTC T-3', inner sense primer (PolF21): 5'-GCA GAC CAG AGC CAA CAG C-3'; *rt5' *segment: outer sense primer (A1): 5'-AAT TTT CCC ATT AGT CCT ATT-3', outer antisense (NE1): 5'-TAT GTC ATT GAC AGT CCA GCT-3', inner sense (NNA): 5'-AAG CCA GGA ATG GAT GGC CCA-3', inner antisense (E): 5'-CCA TTT ATC AGG ATG GAG TTC-3'; *rt3' *segment: outer sense (RTF31): 5'-CCA CAC CAG ACA AAA ARC ATC-3', outer antisense (PolR40): 5'-CTG TTA CTA TGT TTA CTT CT-3', inner sense (RTF32): 5'-CAT CAG AAA GAA CCY CCA TT-3', inner antisense (PolR38): 5'-TTA GCT GCC CCA TCT ACA TAG-3'. PCR products were sequenced by the ABI PRISM BigDye Terminator Cycle Sequencing Ready Reaction Kit (Version 3.1) (Applied Biosystems). The sequenced products were resolved and analyzed using an ABI PRISM 3100 or 3130 *xl *Sequence Detection System (Applied Biosystems) [7]. Relevant positive and negative controls were included in each time to avoid false positive in the PCR.

### Phylogenetic analyses of env, gag and pol genes

Nucleotide sequences from Patients A through D together with other non-clustered control sequences were aligned by ClustalW software [8] and followed by manual adjustment. Phylogenetic and molecular evolutionary analyses were conducted using MEGA version 4 [9]. Evolutionary distances were calculated by Kimura two-parameter modeling, excluding positions with alignment gaps in any sequence. Phylogenetic dendrograms based on partial sequence of the *env C2V3V4 *region, *gag p17/p24 *junction and *pol *gene of the HIV-1 genome were constructed using neighbor-joining method with Kimura two-parameter modeling. The reliability of each node was evaluated by bootstrapping with 2000 replicates.

### Amino acid sequence analysis

*Gag p17/p24 *junction nucleotide sequences from clustered cases were translated into amino acid and aligned with their closest CRF01_AE reference sequence (Accession number: AF197340) and the highest homology matched sequence detected in Yunnan China in 2002 (Accession number: EF062020). Signature analysis was conducted by VESPA tool of Los Alamos National Laboratory  to identify unique sequence pattern present in those clustering cases as compare with the above-mentioned reference sequences [10].

### Statistical analyses

Differences in degree of diversity (evolutionary distance) in *env*, *gag *and *pol *genes of clustered cases and non-clustered controls were ascertained using unpaired *t*-test.

## Results

### Epidemiological investigation

**Figure 1:**
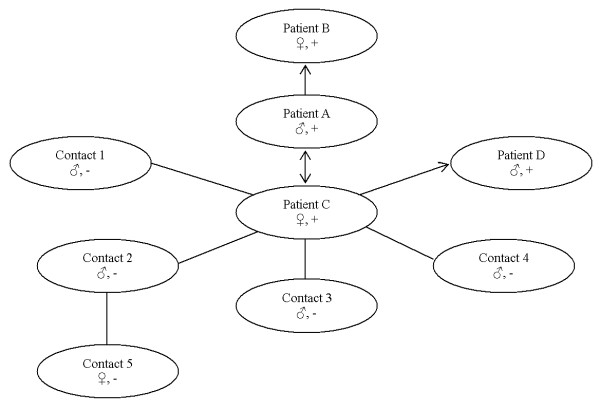
**The relationship between investigated cases and their possible transmission direction**. (Mars male gender symbol) indicates male; (female Venus gender symbol) indicates female; -/+ indicates the result of HIV testing; arrow indicates the possible transmission direction.

In March 2008, the index case (Patient A) died of AIDS 2 months after he was found to be infected by HIV-1. Epidemiological investigation included two females, Patient B (wife of Patient A) and Patient C (sex partner of Patient A). Investigation indicated that Patient B had no other risk factor for HIV-1 infection except for sexual contact with Patient A. Patient C was a CSW since 1998 and served as sex partner of Patient A. Patient C had long term unprotected sex with Patient A in the last 4.5 years and denied any history of drug use, blood transfusion or receipt of blood products. Again, Patient A had denied any history of drug use, blood transfusion or receipt of blood products. Further contact tracing expanded into 5 male sex partners of Patient C, including Patient D and Contacts 1 to 4. Patient D and Contacts 1 - 4 denied risk behaviors for HIV transmission other than heterosexual contact. Patient D served as a sex partner of Patient C for 5 years until he was diagnosed with HIV infection. Contact 1 also was a sexual partner of Patient C for more than 1 year. Contact 2 served as a sexual partner of Patient C two years ago for 1 year. Contacts 3 and 4 had sexual contact with Patient C during the past 4 to 5 years. Another female (Contact 5) who was sexual partner of Contact 2 was included when further tracking of the secondary contacts was conducted. Contact 5 admitted the history of drug use and denied blood transfusion or receipt of blood products. All male individuals in this investigation denied the history of homosexual contact. Patients A through D were confirmed HIV-1 cases, while Contacts 1 through 5 were HIV-1 negative and remained negative after three months follow-up. The relationship between investigated cases and their possible transmission direction is illustrated in Figure 1.

### Phylogenetic analyses

HIV-1 viral RNA was extracted from the sera of Patients A through D. HIV-1 subtype determination using *env*, *gag *and *pol *genes of the HIV-1 genome of these patients consistently showed they belonged to CRF01_AE [7]. Based on the genetic characterization of *gag p17/p24 *junction sequences, it revealed that these four CRF01_AE viral strains were equally homologous (95%) to CRF01_AE isolates detected in Thailand at 1999 (GenBank Accession: AY945731) and Yunnan at 2002 (GenBank Accession: EF062020).

**Figure 2:**
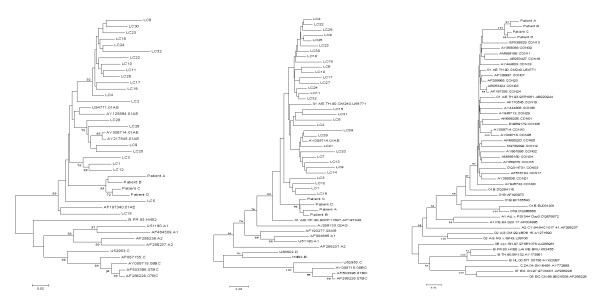
**Phylogenetic analyses of HIV-1 genome of the four patients, controls and reference sequences**. Sequences LC1 to LC32 in *env *tree and *gag *tree indicate randomly selected unrelated CRF01_AE local control sequences. Sequences CON01 to CON30 in *pol *tree indicate randomly selected CRF01_AE control sequences from NCBI GenBank. The HIV-1 reference sequences retrieved from NCBI GenBank. Phylogenetic trees based on the partial sequence of the *env C2V3V4 *region (left), *gag p17/p24 *junction (middle) and a partial of *pol *gene (right) of HIV-1 genome were constructed by using the neighbor-joining method under the Kimura two-parameter model. The number at the node indicates the bootstrap values.

Phylogenetic dendrograms of the acquired *env*, *gag *and *pol *gene sequences from Patient A - D together with randomly selected non-clustered control CRF01_AE sequences were constructed and shown in Figure 2. As expected, phylogenetic analyses indicated that the nucleotide sequences from Patients A - D were clustered together (bootstrap values are 99%, 98% and 100% for *env*, *gag *and *pol *gene respectively).

The evolutionary distances of the *env*, *gag *and *pol *genes from the four patients were 5.23 ± 0.85%, 2.39 ± 0.60% and 1.54 ± 0.22%, and when compared with non-related controls, 11.02 ± 0.92% (n = 23), 6.27 ± 0.66% (n = 32) and 2.95 ± 0.18% (n = 25), respectively (Table 1). The results indicated that the diversity of *env*, *gag *and *pol *genes of these clustered cases (Patient A - D) were significantly lower than non-clustered control group as measured by unpaired *t*-test (*env *gene: *p *= 1.19 × 10^-11^; *gag *gene: *p *= 6.37 × 10^-13 ^and *pol *gene: *p *= 5.12 × 10^-14^).

**Table 1:** Evolutionary distances within the four patients, controls and between patients and controls on *env*, *gag *and *pol *genes ^a^

	***env***			***gag***			***pol***		
			
	***Distance***	***Sample size***	***p-value *^b^**	***Distance***	***Sample size***	***p-value *^b^**	***Distance***	***Sample size***	***p-value*^b^**
Patients ^c^	5.23 ± 0.85	n = 4	1.19 × 10^-11^	2.39 ± 0.60	n = 4	6.37 × 10^-13^	1.54 ± 0.22	n = 4	5.12 × 10^-14^
Controls	11.02 ± 0.92	n = 23	-	6.27 ± 0.66	n = 32	-	2.95 ± 0.18	n = 25	-
Between-group	14.21 ± 1.48	-	-	8.23 ± 1.15	-	-	4.12 ± 0.37	-	-

^a ^Within-group mean nucleotide distances measured as substitutions per 100 sites with estimated 95% standard of error between selected HIV-1 gene sequences. Between-group mean nucleotide distances measured as substitutions per 100 sites with estimated 95% standard error comparing selected HIV-1 gene sequences from the four patients to equivalent sequences from local controls. The control group is randomly selected non-clustered CRF01_AE sequences.

^b ^Unpaired *t-test p-value *calculated with respect to controls.

^c ^Evolutionary distances of all genes are significantly different from the corresponding gene among control sequences (*p *= 1.19 × 10^-11^, *p *= 6.37 × 10^-13 ^and *p *= 5.12 × 10^-14 ^respectively).

### Gag p17/p24 amino acid sequence analysis

**Figure 3:**
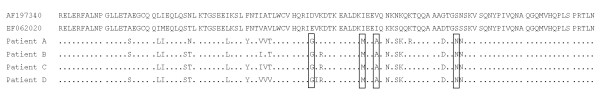
***Gag p17/p24 *junction amino acid alignment**. Amino acid sequences of Patient A - D was aligned and compared with NCBI CRF01_AE reference sequence (Accession number: AF197340) and the highest homology matched sequence detected in Yunnan China in 2002 (Accession number: EF062020). Dots indicate identical amino acid residue. Boxed regions indicate cluster-specific amino acid alterations (frequency = 100%) with respect to AF197340 and EF062020.

Nucleotide sequences of the *gag p17/p24 *junction from the clustered cases were translated into amino acid sequences and aligned with NCBI CRF01_AE reference sequence (Accession number: AF197340) and their high homology sequence detected in Yunnan China (Accession number: EF062020) (Figure 3). The alignment illustrated that clustered cases possess unique amino acid alterations as compared with AF197340 and EF062020. These amino acid alterations were D/E92G, I104M, V/I107A and S124N and formed a specific signature of this cluster.

## Discussion

HIV-1 is characterized by high genomic variability. Sequence diversity was observed whether among isolates within the same individual or between isolates from different infected individuals. Previous studies have shown that HIV-1 isolates from same individual may differ by up to 2% in the *env *gene and those from unrelated individuals may differ by 6-22% [11], while the degree of diversity between isolates from closely related individuals fall in between [12].

Phylogenetic analyses are increasingly used in clarifying the epidemiological linkage of HIV-1 transmission by comparing nucleotide sequence fragments from one or more subgenomic regions, such as *gag*, *env *and *pol *genes, or full-length genome sequence. Early studies have concentrated on sequence variation in the *env V3 *region, which contains the principal neutralization domain and determinants for biological phenotypes [13,14]. Thus, there was argument that it could be less suitable for epidemiological study than other regions (*gag *and *pol*) of HIV-1 genome [15,16]. However, Leitner et al. accurately reconstructed a known HIV-1 transmission history by phylogenetic analysis and demonstrated that it was at least as accurate using *env V3 *sequences as with *gag p17 *sequences [17]. Recent data showed that the *env*, *gag *or *pol *genes were frequently used in epidemiological studies [18-22].

Our evolutionary distance analyses at three different genomic regions (*env*, *gag *and *pol *genes) of the HIV-1 viral genome demonstrated that the HIV-1 isolates from these four patients formed a cluster which was highly related with each other and differed by 5.23% in the *env *gene. Molecular evidence strongly supported the close epidemiological linkage among them. In addition, analyses showed that the average evolutionary distance in *env*, *gag *and *pol *genes of these four clustering members were significantly lower than the ones from local unrelated non-cluster CRF01_AE controls [5,7].

By far bootstrap test is one of the most commonly used tests of the reliability of a constructed phylogenetic dendrogram. The study of Hillis and Bull showed that a bootstrap value of >70% commonly signified a probability of 95% or higher and the topology at this branch is real [23]. Phylogenetic analyses illustrated that the sequences from the four patients clustered together in phylogenetic analyses of both *env, gag *and *pol *genes. The high bootstrap values (99% for *env *gene, 98% for *gag *gene and 100% for *pol *gene, respectively) strongly indicated that they are of closely related and monophyletic.

Amino acid signature was observed in some study to detect for closely related cases, such as transmission of HIV-1 from a dentist to his five patients [5]. The study of a possible single-source sexual transmission cluster in upstate New York showed that a specific amino acid signature was present in the *gag p17/p24 junction *of subtype B sequence [24], while the study of three clusters among subtype B samples in Hong Kong also indicated that amino acid signatures were unique and cluster specific [7]. In this study we detected a unique signature pattern of four amino acid residues in the *gag p17/p24 *junction of CRF01_AE sequences of these four clustering cases in comparison with selected reference sequences.

Genetic analyses of *gag p17/p24 *junction of these four patients revealed that these four CRF01_AE viral isolates were equally homologous (95%) to CRF01_AE isolate which predominated amongst infected heterosexuals in Thailand at 1999 and Yunnan at 2002. It is possible that CRF01_AE strain was transmitted from Thailand through Yunnan China and eventually to Guangzhou China.

## Conclusion

In summary, we performed molecular epidemiology to track the transmission of HIV-1 infection within a group of heterosexual patients in Guangzhou. Highly related sequences from four patients indicated a transmission chain. The result complemented the epidemiological findings that the infection was sustained within the locality through heterosexual contact involving a CSW.

## Sequence Data

Nucleotide sequences of the *env*, *gag *and *pol *gene of Patient A - D were submitted to GenBank at the NCBI (Accession no. FJ752409 - FJ752412, FJ752413 - FJ752416 and FJ752417 - FJ752420 for *env*, *gag *and *pol *sequences respectively).

## Competing interests

The authors declare that they have no competing interests.

## Authors' contributions

ZH carried out the molecular genetic studies. ZH, TWCL and JZ have made contribution to the sequence alignment and drafting the manuscript. MW has contributed to revising the manuscript. LF, KL, XP and ZL participated in acquisition of data and coordination of participants. WLL has contributed to the interpretation of data and critically revised the manuscript. HX conceived of the study, and participated in its design and coordination and revised the manuscript. All authors read and approved the final manuscript.
